# Models of RNA virus evolution and their roles in vaccine design

**DOI:** 10.1186/1745-7580-6-S2-S5

**Published:** 2010-11-03

**Authors:** Samuel Ojosnegros, Niko Beerenwinkel

**Affiliations:** 1 Department of Biosystems Science and Engineering, ETH Zurich, Basel, Switzerland; 2 Present address: California Institute of Technology, Division of Biology, 1200 E California Blvd, MC 139-74, Pasadena, CA, 91125, USA

## Abstract

Viruses are fast evolving pathogens that continuously adapt to the highly variable environments they live and reproduce in. Strategies devoted to inhibit virus replication and to control their spread among hosts need to cope with these extremely heterogeneous populations and with their potential to avoid medical interventions. Computational techniques such as phylogenetic methods have broadened our picture of viral evolution both in time and space, and mathematical modeling has contributed substantially to our progress in unraveling the dynamics of virus replication, fitness, and virulence. Integration of multiple computational and mathematical approaches with experimental data can help to predict the behavior of viral pathogens and to anticipate their escape dynamics. This piece of information plays a critical role in some aspects of vaccine development, such as viral strain selection for vaccinations or rational attenuation of viruses. Here we review several aspects of viral evolution that can be addressed quantitatively, and we discuss computational methods that have the potential to improve vaccine design.

## Review

Viruses are intracellular parasites that need the cellular machinery of the host to reproduce [1]. They have the potential to generate huge population sizes in short generation times. Viruses in general, and RNA viruses in particular, exist in genetically heterogeneous populations because of their error-prone replication [2]. These features make the evolution of viruses a phenomenon observable on short time scales of weeks to months. The consequences of the extreme viral evolutionary dynamics are of tremendous importance for disease control and prevention. For example, influenza vaccines need to be updated every year, viral variants develop resistance to antiviral drugs, and mild viral strains turn into virulent ones spontaneously. These global health care issues and others arise from the rapid evolution of viruses. 

Molecular profiling techniques, including DNA sequencing, have produced an enormous amount of data on viral spread, genetic diversity, and infection dynamics. The integration and analysis of these data can provide valuable information on the evolution of viral pathogens. Mathematical, statistical, and computational methods are necessary to deal with those large data sets and to predict phenotypes form genetic data that ultimately can be used in vaccine development. In this review, we summarize some computational and mathematical techniques that play a critical role in understanding viral evolution and vaccine design. Specifically, we discuss phylogenetic methods for vaccine strain selection, statistical models of evolutionary escape from selective immune pressure, and virus dynamics models for therapeutic vaccines and attenuation strategies. Our major examples are Influenza, human immunodeficiency virus (HIV), and foot-and-mouth disease virus (FMDV). They are all RNA viruses of great medical or veterinary importance and have been studied extensively.

We also discuss current, approved experimental methodologies employed to the development of antiviral vaccines. Vaccine development deals with the four most important traits of viral evolution: virulence, fitness, diversity, and dynamics. Each one of these concepts has been subjected to intense studies but is still difficult to predict, probably because of the complexity of each trait and the intrinsic stochasticity of the evolutionary process. Progress in understanding the interplay of these factors will depend on quantitative descriptions and predictive models. Thus, mathematical evolutionary modeling is likely to play an increasingly important role in the development of new vaccines and the control of viral disease.

### Viral evolution

A quantitative description of viral evolution is necessary for monitoring the spread of viral pandemics and for developing effective therapies and vaccines [3]. Viruses are not only a threat to human health, but they also provide attractive model systems for evolutionary studies due to their short genomes, large population sizes, and high genetic diversity [4]. The extreme replication dynamics of RNA viruses, for example, allow for observing significant evolutionary changes over time. Hypotheses and theories about evolutionary mechanisms can often be tested directly with these measurably evolving viruses [5].

The mutation rate of RNA viruses is about a million times larger than the human mutation rate [6]. Thus, RNA viruses display a huge genetic diversity and this feature is critical for survival of the virus [7,8]. Virus populations are exposed to fluctuating environments when migrating through different organs and tissues of the host organism and when exposed to immune responses mounted by the host. Transmission to a new host is typically associated with both traversing various tissues and facing new immune responses, and therefore it represents a major bottleneck for the virus population. The genetic diversity of RNA viruses makes it likely that adapted variants preexist in the population even before the selective pressure has changed [9,10]. 

Because the diversity of the virus population can determine its evolutionary fate, selection seems to operate on the population level rather than at the level of individual viruses [11]. This idea was originally developed for self-replicating RNA molecules and termed quasispecies theory [12], and then applied to RNA viruses [13,14]. One prediction of quasispecies theory is the existence of an upper bound on the mutation rate beyond which the population cannot maintain its essential genetic information. Many RNA viruses appear to have mutation rates close to this error threshold [9,15,16].

The evolutionary dynamics of viral infectious diseases can be analyzed at considerable detail today owing to advancements in high-throughput DNA sequencing technologies and statistical and computational modeling of these data [17]. Viral evolution occurs on different temporal and spatial scales and is shaped by different ecological processes within and between hosts. Integrated modeling efforts across these scales that make use of phylogenetic, population genetics, virus dynamics, and epidemiological methods are termed phylodynamics approaches [18]. Application of these techniques enabled the reconstruction of the molecular origin of the HIV pandemic [19,20] and the explanation of influenza A epidemics by the interplay of natural selection and migration [21,22]. Comparing viral DNA sequences is at the heart of the phylodynamics approach.

In general, the study of correlated evolution among genotypic and phenotypic traits or between traits and the environment across species is known as the comparative method [23]. It has been extremely successful in analyzing DNA sequence data and it is the basis for predicting phenotypes, such as protein structure or function, from genotypes. In the case of RNA viruses, which display a large genetic diversity between hosts, the different viral quasispecies take the role of the different species in the traditional application of the comparative method. Viral phenotypes of interest include immunological escape and drug resistance. 

### Vaccine development strategies

Vaccine development can be considered one of the biggest achievements of modern medicine. While some bacterial families share common biochemical features, such as surface lipopolysacharide and therefore can be treated with common drugs or antibiotics [24], viruses are typically different enough between families or even inside families, making the development of broadly applicable antiviral drugs challenging [2,25]. For example, the base analogue ribavirin alone or in combination with interferon, has been proven to act as an antiviral compound of broad activity in the clinical treatment of infections with hepatitis C virus (HCV), hepatitis B virus (HBV), and respiratory syncytial virus (RSV) [26-31]. Overall, only few antiviral drugs targeting viral proteins are currently approved for use in humans [32]. Viruses that can be fought with chemical compounds include HIV, influenza virus, RSV, HCV, HBV, and the herpes viruses, including herpes simplex virus, varicella-zoster virus (VZV), and cytomegalovirus. 

However, currently the vast majority of viruses cannot be controlled today with any approved compound and frequently not even all subgroups inside the same viral species are drug sensitive. For example, the 2009 seasonal influenza A virus (H1N1) presents a natural resistance to oseltamivir [33]. The efficiency of antiviral compounds is usually hampered by the generation of drug resistant viruses. Moreover, some compounds may help control infection, but only rarely can the virus be completely cleared from the host organism. By contrast, vaccines can boost the immune system response and, in principle, achieve complete clearance of a virus from an infected host. Therefore, vaccines are considered the best weapon to fight viruses.

The general idea of vaccination is to resemble a viral infection but avoiding the associated pathology. There are several strategies to address this goal with varying efficacies. Currently approved viral vaccines for use in humans are listed in Table 1. They have resulted from one out of three main types of vaccine design strategies referred to as (*i*) live attenuated vaccines, (*ii*) inactivated vaccines, and (*iii*) recombinant vaccines and peptide-derived vaccines.

**Table 1:** Vaccine-preventable viral diseases as defined by CDC Atlanta [175].

Disease	Virus	Type of vaccine
Viral hepatitis	hepatitis A	inactivated
	hepatitis B	recombinant (subunit, surface antigen)
Flu	influenza	inactivated
Mumps	mumps virus	live attenuated, MMR vaccine^1^,
Measles	morbilivirus	live attenuated, MMR vaccine^1^
Polio	poliovirus	live attenuated, Sabin strain
		inactivated, Salk strain
Rubella	rubella virus	live attenuated, MMR vaccine^1^
Cervical cancer	human papillomavirus	inactivated
Japanese encephalitis	Japanese encephalitis virus	inactivated
Children severe diarrhea	rotavirus	live attenuated
Rabies	rabies virus	inactivated virus
Smallpox	smallpox	live attenuated vaccinia virus^2^
Varicella (chickenpox, shingles), herpes zoster	varicella-zoster virus	live attenuated
Yellow fever	yellow fever virus	live attenuated

^1^ MMR is a triple vaccine including Mumps, Measels, and Rubella vaccine.

^2^ Small pox vaccine is no longer routinely administered because of eradication of smallpox. The vaccine consists of a stock of vaccinia virus, a smallpox-related virus which does not cause disease in humans.

*Live attenuated vaccines (LAVs).*  Immunization with LAV has been proved to be the most efficient vaccination strategy to date [34,35]. LAV preparations include viruses with reduced virulence, which means that they do not produce the disease when infecting the host, or they produce a mild version of the disease. Viruses become attenuated for the original host after serial infections (passages) in cell culture of different organism. This is the case of the polio vaccine preparation in monkey cells [36]. Infection of embryonated hen eggs is the standard protocol to obtain attenuated yellow fever or measles viruses suitable for vaccine preparation [37]. The rationale for this attenuation strategy is that due to the high error rates during replication of viruses, especially of RNA viruses [2], the virus accumulates mutations in the genome that optimize their replication in the new host or new cell type, at the expense of replication efficiency in the original host [1,34,38].

The attenuated virus is still competent for replication and it retains the ability to infect host cells. For this reason, LAVs can elicit different effector mechanisms of the immune system. The intracellular replication of the virus can stimulate cytotoxic CD8^+^ cells because they can be presented by major histocompatibility complex (MHC) class I molecules. Particles released outside the cell can also be presented by class II MHC molecules [39]. After immunization with the LAV, the immune system is exposed to multiple antigens of the virus in its native conformation. Once the infection with the LAV is cleared from the organism, the virus-specific immune cells remain as memory cells in the host. A future challenge with the wild type virus will trigger the correct response, i.e., predominantly cellular response or predominantly humoral response. LAVs are considered the most successful vaccines because the efficient and multiple stimulation of the immune system typically induces a potent and durable response [38]. 

*Inactivated vaccines (IVs).*  Viral stocks are susceptible to inactivation by some chemical and physical treatments. IVs consist of a concentrated viral stock that has been treated with a chemical reagent, such as binary ethylenimine or formaldehyde, which completely abolish virus replication [40]. Some viruses are difficult to attenuate because they may change their antigenic properties or they remain virulent after few passages in cell culture [41]. The latter is the case for FMDV, an animal virus from the picornavirus family. When a virus cannot be attenuated with sufficient reliability, inactivation has been proven to be a successful vaccination strategy, as documented for FMDV, influenza, or hepatitis A vaccines [41-44]. Some security issues may arise however, if the chemical compound does not reach all virus particles, for example because some viral particles tend to form compact aggregates, and the preparation still contains a portion of live viruses. Inactivated viruses are usually not efficiently presented by MHC class I molecules, which stimulate the cellular immune response. IV preparations include adjuvants which are chemical compounds that act on antigen-presenting cells enhancing the immunogenicity of the vaccine [45,46]. However, the strength and duration of the protection induced by IVs is usually lower than that obtained with LAVs.

*Recombinant and peptide-based vaccines.*  A great variety of vaccine strategies can be catalogued inside this category, most of them experimental. Recombinant vaccines are produced by the expression of a genetic construct that codifies viral peptides [47,48], subunits of the virus [49], or whole viruses with genetic modifications including deletions of key proteins [50,51]. Other strategies include live viral vectors that carry multiple copies of heterologous proteins of interest [52,53]. Many of these strategies have failed, mainly because of the low immunogenic capacity of peptides or subunits, compared with the whole live or inactivated particle. The HBV vaccine is a yeast-derived recombinant vaccine. It contains the hepatitis B surface antigen which is one of the viral envelope proteins. HBV vaccine is the only recombinant vaccine currently approved and in use for humans [49].

DNA vaccination is a strategy based on injecting a DNA construct directly into the host [54,55]. Such DNA constructs, which code for the immunizing protein or other parts of interest of the virus, can be transcribed and translated into the cell. Therefore, expressed gene products can elicit an immune response by presentation of peptides by MHC class I and II molecules. 

### Phylogenetic methods in influenza vaccine design

Influenza A virus is a negative-stranded RNA virus that infects about one fifth of the worldwide human population each year [56]. The viral genome consists of eight segments and is categorized by the serology and genetics of its two surface glycoproteins neuraminidase (NA) and hemagglutinin (HA). Several subtypes of both genes have been isolated from mammalian and avian hosts, including the two most recent pandemic strains H3N2 and H1N1 currently circulating in the human population and responsible for the 1968–1969 Hong Kong Flu and the 2009 Swine Flu, respectively.

Influenza infects large portions of its host population every season and immunized hosts are resistant to infection with the subtype they have been exposed to for several years. Therefore, selective pressure exists for the virus to diversify and to generate immunological escape variants. Indeed, the HA gene has been shown to be under strong selective pressure through immune surveillance [57]. Positive (diversifying) Darwinian selection acts at the antigen-determining sites of HA1, the most immunogenic part of HA. At these loci, significantly more non-synonymous than synonymous nucleotide substitutions are observed, and the rate of evolution is accelerated considerably as compared to other sites of the genome [58,59].

Not only selection, but also neutral genetic drift seems to play an important role in the evolution of influenza virus. Both evolutionary forces, termed antigenic drift and antigenic shift, have been observed in human hosts over the last century. Antigenic drift refers to neutral evolutionary changes accumulating over time, whereas antigenic shift involves a change in genetic and serological properties of the virus due to new HA or NA subtypes. 

The evolutionary dynamics of H3N2 epidemics have been studied in detail by allowing for different genetic and antigenic properties of the HA gene [60]. Variations of this influenza subtype emerge and replace each other every 2 to 8 years. The mapping of HA genotypes to antigenic phenotypes is based on a neutral network model, i.e., mutants are organized in connected, phenotypically identical clusters. The model explains the observed pattern of viral evolution, with periods of antigenic stasis and with episodic cluster transitions. During antigenic stasis the population drifts through the neutral network and its diversity increases. If it hits the boundary of the neutral network, the antigenic phenotype changes and the population continues to evolve in the new network. The cluster transition presents a population bottleneck and gives rise to a selective sweep which reduces the genetic diversity of the population [61].

The influenza vaccine needs to be redesigned regularly to account for genetic changes in the virus population. Normally, the changes are made in response to the antigenic drift of the virus. For example, between 1968 and 2001 the H3N2 component of the influenza vaccine was changed a total of 17 times [62]. The selection of viral strains to be included in the vaccine for the coming season is based on the antigenic properties of recent isolates, on epidemiological data, and on post-vaccination serological studies in humans. 

The evolutionary dynamics of influenza drive its immune escape and give rise to a new dominant strain every season. Therefore, vaccine design is not only supported by immunoinformatics methods for epitope prediction [63-65], but also by statistical genetics and phylogenetic methods for analyzing genetic diversity and predicting evolutionary changes. To predict the evolution of the influenza HA gene, phylogenetic trees were constructed based on DNA sequences derived from viruses during the years 1983 through 1997 [22]. Eighteen codons were identified to be under positive selective pressure and the genetic diversity at these loci was significantly higher than at the other loci of the HA gene [59]. The rationale for predicting the next dominant virus is that extant strains with additional mutations at the 18 loci will be better adapted to evade the host immune response and thus have a selective advantage in the coming season. Phylogenetic analysis confirmed that the viral lineages with the greatest number of mutations in the positively selected codons were the ancestors of future H3 lineages in 9 out of 11 influenza seasons [22].

This approach to predicting the evolution of influenza relies on solving two classical evolutionary biology problems: the detection of genetic loci under selective pressure and the reconstruction of the evolutionary history of a set of individuals. Quantifying the relative contributions of selection versus random genetic drift is a longstanding task rooted in Kimura’s theory of neutral evolution which predicts that most mutations are selectively neutral [66,67]. Selection is typically identified by testing for departure from neutrality, although such deviations can also have different causes. The statistical tests are either based on the allelic distribution or on comparing variability in different classes of mutations [68]. Tajima’s *D* is the prototype test of the first kind [69]. It detects differences in two distinct estimates of genetic diversity. The null distribution of the test statistic *D* is obtained from sampling genealogies according to the coalescent, a stochastic process describing the sampling variation [70,71]. Similarly, the Ewens–Watterson test compares the observed to the expected homozygosity based on the Ewens sampling formula for the infinite-alleles model [72,73]. In the second category of tests fall the McDonald–Kreitman test [74] and likelihood ratio tests based on the allelic distribution in non-synonymous versus synonymous sites [58,75]. Codon usage in influenza sequences has also been analyzed based on codon volatility, which measures the degree to which a random nucleotide mutation is expected to change the corresponding amino acid [76].

Phylogenetic methods play an important role in the analysis of viral sequence data. They are used to reconstruct the evolutionary relationships between different viral strains. Three main approaches exist to phylogenetic tree reconstruction: distance-based methods, parsimony, and probabilistic methods based on maximum likelihood or Bayesian statistics [77-80]. Distance-based methods start by defining an evolutionary distance between sequences and then apply hierarchical clustering algorithms to obtain a phylogenetic tree. Unweighted Pair Group Method with Arithmetic mean (UPGMA) [81], an average linkage clustering method, and Neighbor Joining [82] are two prominent examples of this approach. Distance-based methods are computationally efficient, but the reduction of the observed data to a distance matrix presents a loss of information. Both UPGMA and Neighbor Joining can reconstruct the correct tree only under strong assumptions about the metric defined by the distance matrix and they are sensitive to violations of these assumptions.

Unlike distance-based methods, character-based methods follow character substitutions in the sequence explicitly. Maximum Parsimony is based on the minimum evolution principle and tries to find the tree that explains the data by the minimum number of mutations [83]. The method has been applied successfully to the analysis of influenza virus sequence data [84].  It is also computationally efficient, but lacks an explicit evolutionary model (other than minimum evolution), and it is not statistically consistent. On the other hand, probabilistic phylogenetic models have these desirable properties, but they come at a computational cost usually rendering exact maximum likelihood estimation impossible. However, approximate likelihood methods, such as DNAML [59] or QuartetPuzzling [85], and methods making use of Bayesian inference, such as MrBayes [86] are applied in practice. For both distance-based methods and probabilistic phylogenetic methods, the choice of an appropriate model of nucleotide substitution is critical.

The application of statistical genetics and phylogenetics methods to influenza sequence data has not only improved our understanding of the evolutionary dynamics and the epidemiology of the virus, but it has also become an integral part of the yearly vaccine design cycle. However, the successful case of influenza does not seem to provide a practical model for HIV. One reason for this discrepancy might be the evolutionary dynamics of HIV which are strikingly different from those of influenza. Rather than the drift-and-shift pattern of influenza evolution which generates only a small amount of genetic diversity around the successful trunk lineage, HIV tends to spread out from an ancestor in a radial fashion and to generate much more variation. The worldwide diversity of influenza sequences in any given year appears to be comparable to the diversity of HIV sequences found within a single infected individual at one time point [62]. Thus, an HIV vaccine must stimulate a very broad reactive immune response against a large set of diverse viral strains and the genetic makeup of these sequences is much more difficult to predict from the currently circulating strains as compared to influenza. It is for these and possibly other reasons that the same bioinformatics-assisted vaccine design approach that is established for influenza, has not been equally successful for HIV to date. In the following sections, we will discuss extensions of the models discussed above as well as complementary mathematical and computational approaches that might be of help in search for an HIV vaccine in the future.

### Evolutionary escape of HIV from selective immune pressure

HIV populations display a high genetic diversity due to the quasispecies nature of RNA virus replication [2,87,88]. HIV occurs in three main groups, the principal of which, group M, is composed of nine subtypes. Moreover, HIV has a great capacity for recombination among subtypes, and as a consequence, new circulating recombinant forms are constantly arising [89,90]. Each subtype itself represents a great genetic diversity as well and even during infection of a single patient, HIV exists as an ensemble of different sequences [88]. The high genetic diversity of HIV populations hampers the development of a vaccine of broad applicability. Vaccine candidates need to elicit responses against multiple epitopes in order to counteract the immune evasion by virus mutation [91,92].

With one exception, the few attempts to bring HIV vaccines to the last phases of clinical trials have been quite disappointing so far. One of the first and most prominent vaccine candidates, the VAXGEN vaccine, was intended to immunize subjects with a recombinant envelope protein of HIV (rgp120) [93,94]. The envelope protein is located on the surface of HIV and is responsible for the attachment of the virus to the host cell surface receptor [95]. Antibodies targeted against this protein could block HIV infection and subsequently block virus entry into cells. During VAXGEN trials, the immunization induced the production of antibodies in vaccinated individuals, but they were unable to control infection or viremia. 

The STEP vaccine trials were designed to test the efficiency of a T cell vaccine in reducing viremia and enhancing the cellular immune response [96]. This vaccine candidate is a therapeutic vaccine (see below), because it was intended to enhance the immune response against HIV even if sterilizing immunity would not be achieved. The STEP vaccine formulation was based in an adenovirus serotype 5 vector (Ad5). The vaccine included three independent Ad5 vectors, each one carrying one of the three HIV proteins Gag, Pol, and Nef. Although in early phases of the clinical trials, the vaccine was proven to elicit specific anti-HIV T cell responses, no significant protection was conferred to people receiving the vaccine in phase IIb trials. Indeed, individuals having immune memory against the Ad5 vector were more susceptible to infection by HIV [97]. This unexpected result was probably due to an adding-fuel-to-the-fire effect, in which the Ad5 vector activated T cells creating a suitable environment for HIV replication [98].

Recently, a combined vaccine of recombinant canarypox vector vaccine [99] plus a recombinant glycoprotein 120 subunit vaccine has been tested in phase III clinical trials in Thailand involving more than 16.000 subjects. The results showed a slight trend of protection in the vaccinated group when compared to the control group. Although these numbers are still debated [100] and vaccination did not affect the degree of viremia or CD4+ T cell count in HIV infected subjects, the results offer insight for future research.

In order to address the challenge of HIV sequence diversity, several new vaccine design strategies are explored based on combining different epitopes. These methods include the construction of pseudo-protein strings of T cell epitopes [101] and the synthetic scrambled antigen vaccine, which combines consensus overlapping peptide sets from HIV-1 proteins [102]. Both approaches select codons, peptides, or sequences according to codon usage, covered diversity, and predicted HLA affinity, and randomize sequence fragments.

Vaccine design strategies based on whole viral protein sequences make extensive use of phylogenetics. In addition to the basic methods discussed above, HIV-specific probabilistic models of protein evolution have been constructed which allow for improved phylogenetic inference using likelihood or Bayesian methods [103]. The reconstructed phylogenetic tree can guide the selection of viral genomes to be included in the vaccine. Different selection strategies have been proposed to stimulate a broad immune response and to minimize the amount of sequence divergence between the antigen and contemporaneously circulating viruses. Natural strains that represent the total observed sequence space or derived consensus sequences have been selected as vaccine strains [104-106]. Probabilistic phylogenetic models also allow for inferring the DNA sequences at internal nodes of the tree which represent extinct common ancestors. The most recent common ancestor (MRCA) of a given set of viruses is the root of the phylogenetic tree for these sequences [107]. It has been proposed as a vaccine strain stimulating cross-reactive immune responses against all of its descendants [104,108]. However, in asymmetric phylogenies, both the consensus sequence and the MRCA can perform poorly at minimizing the distance to contemporary strains [109]. To address this limitation, the center of tree node has been proposed. It is calculated as the node minimizing the least squares distance to all leaves of the phylogenetic tree [110].  

The usefulness of phylogenetic trees is limited in the presence of reticulate evolutionary events, such as hybridization, horizontal gene transfer, or recombination, which cannot be represented by a tree. For this situation, phylogenetic network models have been developed [111]. They generalize phylogenetic tree models and include reticulate networks and split networks [112]. In most RNA viruses, homologous recombination can occur when a cell is coinfected with two different strains. In HIV, multiple infections are common [113] and the recombination rate is on the order of 2 to 3 times per genome per replication cycle [114]. Several epidemiological circulating recombinant forms provide evidence for recombination in HIV. Intra-host evolutionary dynamics are also shaped by recombination affecting the generation of multidrug-resistant strains in treated patients [115,116] and the development of immune escape variants. Efficient parsimony algorithms have been developed for computing recombination networks [117,118].

Immune responses to HIV infection vary depending on the genetic constellation of the human leukocyte antigen (HLA) locus. Antigen-specific T cell immunity is HLA-restricted and therefore mutations in HIV epitopes that allow escape from host immune responses are HLA allele-specific [119,120]. Cytotoxic T lymphocyte (CTL) escape mutations have been shown to be stable under vertical transmission of the virus [121]. Thus, CTL escape presents an important driving force in shaping HIV evolutionary patterns. Differential HLA-restricted viral evolution has been observed in several HIV-1 genes [122]. From a vaccine design point of view, it is pivotal to characterize CTL escape quantitatively in order to address limitations of immune stimulations and to minimize the risk of viral evolutionary escape. This task involves identification of HLA-specific escape mutations and prediction of mutational escape pathways.  

HIV polymorphisms associated with CTL escape leave HLA-specific footprints at the population level and hence detection of escape mutations is based on the comparative method. However, there are at least three confounding factors in this approach. First, viral sequences obtained from different hosts cannot be regarded as independent observations of the same stochastic process. Rather, these sequences share a common evolutionary history. Second, structural and functional limitations, such as the biophysics of three-dimensional protein structures, impose constraints on codon covariation and give rise to linkage disequilibrium (LD) in the HIV genome. Third, the host’s HLA genes are also in LD because of their close physical distance on human chromosome 6. 

Phylogenetic dependency networks (PDNs) are a class of probabilistic graphical models that account for these confounding factors [123]. PDNs explicitly represent selection pressure from multiple sources and model the dependency structure among escape mutations conditioned on a phylogenetic tree of the observed viruses and on the HLA types of their hosts. By analyzing 1000 individuals from a multicenter cohort, the statistical model identified a dense network of interactions between HLA alleles and HIV codons, as well as among HIV codons, reflecting the complexity but also the consistency of HIV adaptation to immune response [123].

**Figure 1:**
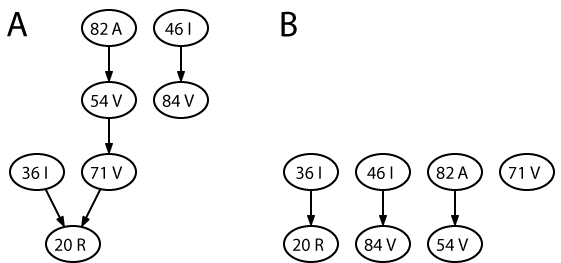
**Conjunctive Bayesian networks describing HIV evolution under therapy with the two protease inhibitors ritonavir (A) and indinavir (B).** The vertices of both graphs correspond to the same drug resistance-associated amino acid substitutions K20R, M36I, M46I, I54V, A71V, V82A, and I84V, in the HIV-1 protease, where K20R stands for a change from lysine (K) to arginine (R) at position 20, etc. Directed edges of the graphs denote partial order relations that constrain mutational pathways. An edge X → Y indicates that mutation Y can only occur after mutation X has occurred. The H-CBN program from the CT-CBN software package [174] has been used to generate the models from 112 and 691 samples for ritonavir and indinavir, respectively.

HIV mutational pathways have also been modeled in the context of evolutionary escape from the selective pressure of antiretroviral therapy. Several probabilistic models have been developed for describing the accumulation of amino acid changes in response to specific drugs or drug combinations, including Markov chains [124], Bayesian networks [125], mutagenetic trees [126-128], and conjunctive Bayesian networks (CBNs) [129]. CBNs are a class of probabilistic graphical models that describe the order in which mutations occur. In this model, the partial order of mutations that maximizes the likelihood of the data can be learned efficiently from observed mutational patterns. CBNs allow for several escape paths with different probabilities and the partial order restricts the viral genotype space to the subset of those mutational patterns compatible with the partial order constraints (Figure 1). The rationale for this approach is that many combinations of mutations are never observed, for example, because they result in inviable viruses, or because they are too far away in sequence space from current strains to be reached on the relevant time scale. CBNs can explicitly represent the timeline of mutations occurring along escape pathways [130] and they have been extended to account for noisy observations [131].  

Computational models of viral escape dynamics have been applied successfully in the design of optimal combination therapies [132,133]. Because the development of drug resistance is a major factor for treatment failure, not only the current resistance profile, but also the likelihood of evolving resistant viruses is a strong predictor of therapeutic outcome. The difficulty for the virus to escape from the applied selective drug pressure is known as the genetic barrier and it can be computed based on probabilistic models of accumulating mutations [127]. Retrospective analyses of large observational clinical databases have demonstrated that estimates of the genetic barrier based on viral progression models are independent predictors of treatment outcome. The genetic barrier improves therapy outcome predictions and the resulting models outperform standard-of-care expert rule-based treatment recommendations [134,135]. Therefore, computational models of viral escape dynamics might also be useful for vaccine design. A successful HIV vaccine should not only minimize the distance to currently circulating strains, but also anticipate possible immune escape pathways of the virus. Although it is unlikely that the complete picture of escape pathways can be learned from data, improvements in terms of hindered and delayed escape might be possible, especially in the context of therapeutic vaccines where selective immune and drug pressure together may constrain virus evolution significantly and result in control of infection. 

Besides genetic heterogeneity, the development of an efficient vaccine against HIV remains elusive because of the difficulties of inducing an efficient immune response [98,136]. Individuals who control infection display a strong cellular response [137-139]. Many experimental vaccines have failed in directing the effector response to a more cellular profile [140]. Furthermore, HIV infection induces a low titer of neutralizing antibodies [141,142]. Gp120 and gp41 are the HIV proteins exposed at the surface of the virion. These proteins are responsible for the attachment of HIV to the cell surface and the virus has developed several strategies to avoid recognition and blocking by antibodies. The region of the protein that interacts with the CD4 cellular receptor is a hypervariable loop. A great number of antibody-escape mutants are mapped to this region of the HIV genome. The loop however, is highly glycosylated and it is only exposed at the surface of the protein in the precise moment of the interaction with the cellular receptor [143]. These two combined features complicate the fitting of potentially neutralizing antibodies [142].

HIV is a member of the retrovirus family with the ability to integrate its genome into the host cell genome [95]. Genome integration is another challenge to develop an effective vaccine because latently infected cells cannot be recognized by the immune system until the integrated provirus is activated [136].

After several attempts to obtain a protective vaccine against HIV, current efforts have shifted toward developing therapeutic vaccines which help to control infection. A vaccine that elicits an incomplete response may be sufficient to keep viral load in controllable levels. The vaccine would not prevent infection by HIV, but it would delay or prevent the progression to AIDS, the final stage of the disease [137]. The reduction in viral load would also reduce the number of secondary transmissions of the infection, because the efficiency of transmission depends on viral load levels.

### Virus dynamics and therapeutic vaccines

**Figure 2:**
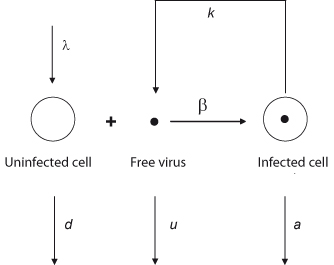
**Schematic diagram of the basic model of virus dynamics.** When a susceptible cell and a virus meet, the cell becomes infected. The infected cell releases to the extracellular medium the progeny of the initial infecting virus. The new progeny will in turn infect additional susceptible cells. At this point a chain reaction is started which is the basis of the cellular and viral dynamics during an infection (adapted from [144]).

A viral infection is a complex molecular process, but it can be approximated by a few major steps (Figure 2). Initially, the virus needs to find a susceptible cell and enter into it. Once inside the cell, virus replication starts and viral offspring is released. Infected cells will eventually die. Released virus particles are either inactivated or they hit a new susceptible cell, in which case the infection cycle is closed and a chain reaction of sequential infections is triggered [144-146]. The dynamics of the viral replication cycle can be expressed in mathematical terms as follows:

This ordinary differential equation (ODE) system describes uninfected cells, *x*, being infected with efficiency *β*, infected cells, *y*, dying and releasing viral offspring at rate *a*, and free virus, *v*, being produced at rate *k* and inactivated at rate *u*. In the absence of viral infection, cells reproduce at rate *λ* and die at rate *d*. Oversimplifying the role of the immune system, the immune cells, *z*, grow and die with rates *c* and *b*, respectively. They remove infected cells from the system with efficiency *p*. Each specific viral family may give rise to modifications of this model due to variations in its life cycle. But the ODE system is the core of a family of mathematical models that describe the turnover of viruses and cells during an infection. 

**Figure 3:**
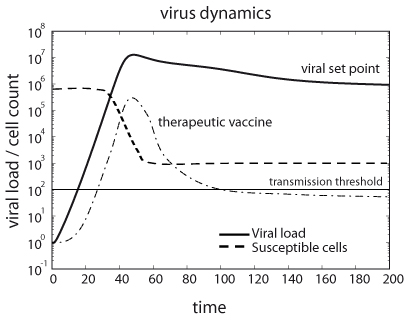
**Dynamics of viral load and susceptible cells before onset of the immune response.** Initially, viral load grows exponentially to eventually achieve an equilibrium termed viral set point. Therapeutic vaccines might slow down the initial exponential increase of viral load, which consequently implies a reduction in the viral load during the set point phase of the infection. The plot represents a typical chronic infection such us the one produced by HIV.

Virus dynamics models have been successfully employed to the study of simian immunodeficiency viruses (SIV), HIV, and HBV, among others [144,146-150]. The dynamics of this model are shown in Figure 3. The model is an epidemiological SIR model [151], in which infection is treated as a microepidemic and host cells play the role of infected or susceptible individuals. Whether the virus infection can spread in the cell pool or not depends on a condition very similar to the spread of an epidemic in a population of individuals. The parameters of the model must satisfy the inequality *R*_0 _> 1, where *R*_0_ is the basic reproductive number, defined as the number of newly infected cells that arise from any one infected cell when almost all cells are uninfected [144,151].  For *a*’ = *cp*/*b*, this number is given by

For generic parameters, if *R*_0_ > 1 uninfected cells become infected and produce progeny viruses exponentially. Activation of the immune system (*a*’ > 1) reduces the value of *R*_0_ and slows down the spread of the infection. At the beginning of the infection, before the immune response is mounted (*a*’ ≈ 0), and after the initial peak of viral load, viruses and infected and uninfected cells reach a stable equilibrium termed viral set point (Figure 3). While monitoring viral load of SIV in infected macaques, a correlation between the viral load at initial stages of the infection and the viral set point was observed [152,153]. One can demonstrate that the equilibrium abundance of viruses, v*, and the logarithm of the virus load during the exponential growth phase, follow the linear relationship

This result is important in HIV research, because several studies indicate that there is a positive correlation between viral load and disease progression. Individuals who display a lower viral load during the first stage of the infection have higher chances to survive and control the infection [154].

From the virus dynamics point of view, a successful vaccine is one that boosts the immune response, i.e., increases the parameter *a*’, such that *R*_0_ is reduced below the critical value of 1. Under such conditions, the infection will initially grow, but then viral load will rapidly decline and the infection will eventually be cleared from the organism.  However, even if the vaccine induces an immune response that is not strong enough to reduce *R*_0_ to a value below 1, it may still be beneficial. As described above, slowing down the initial exponential increase of the viral load has a negative effect on the viral set point. This type of imperfect vaccine is also called a therapeutic vaccine. In the case of HIV, it could help infected patients to control disease progression in two ways. First, it may reduce viral load during the chronic phase of the infection, and second it may reduce viremia below the threshold of inter-host virus transmission (Figure 3) [137,144,155-157].

On the epidemiological level, mathematical models of inter-host transmission of a given viral pathogen can predict the portion of individuals within a population that needs to be vaccinated in order to achieve herd immunity (HI). HI occurs if partial vaccination provides protection also to unvaccinated individuals and the population is protected against invasion of the pathogen. The critical proportion of immune individuals above which the disease cannot persist is the HI threshold

Here *R_0_* is the basic reproductive number of the pathogen in the host population, rather than in the cell population of a single host as discussed above. The higher the reproductive ratio of the disease, the more individuals need to be effectively protected by vaccination. The HI threshold varies not only with the virulence of the disease, but also with the efficacy of the vaccine and the contact dynamics of the host population.

### Attenuation strategies and the evolution of virulence in RNA viruses

Individual viruses inside the quasispecies can display very different degrees of virulence. This trait is measured as the cell killing capacity, when considering cell culture experiments, and as the host morbidity or mortality, when focusing on whole-organism pathology [158,159]. Replicative fitness is also highly variable among viruses of the same quasispecies, and in general, not directly correlated to virulence [158]. Understanding the evolution of these two viral traits is fundamental to understanding and controlling the spread of viral diseases and to the design of LAVs.

Since attenuated viruses may elicit a potent immune response without causing harm to the host, several strategies are being explored to obtain candidate viruses for LAVs, i.e., viruses displaying a reduced cell killing or replicative ability. Serial cytolytic transfers in cell culture tend to select viruses attenuated in the original host [160]. This is the case for FMDV or yellow fever virus among other viruses, and is currently an approved technique for several LAV preparations. Viruses selected after severe bottlenecks, such as serial plaque-to-plaque transfers, present a reduced fitness due to the accumulation of mutations associated with the Müller’s ratchet effect [161,162]. A new promising strategy for the attenuation of viruses is the rational design and synthesis of viral genomes with a strong codon bias. This approach has been implemented for Poliovirus and influenza virus [163,164]. The viral genome synthesized encodes the same amino acid sequence as the wild type virus but encoded by infrequent codons in their host cells. Viruses harboring fidelity mutations in the replicase genes tend to produce a quasispecies of lower diversity and to be attenuated *in vivo*. This feature has also been employed to the rational design of a Poliovirus LAV [165]. Other strategies conceived to limit viral replication include the design of specific microRNAs or zinc finger nucleases targeting the viral genome [166].

Many mathematical models have been developed to describe the evolution of virulence in diverse viral populations. One conclusion of these theoretical studies is that virulence can increase in the population under a variety of conditions [167]. The basic model of virus dynamics, however, states that less cytopathic variants are more productive in the long term of the infection, because the abundance of both viruses and infected cells is inversely correlated with the cytopathogenicity, *a*, of the viral variants [144]. For *R*_0_ > 1, 

Recently, a FMDV population has been reported to diversify into two genetically distinct subpopulations that also differ in virulence. The viral variants have been characterized phenotypically in considerable detail and their coevolutionary dynamics, when competing for the same cell pool *in vitro*, have been analyzed. The competition experiment has been described by an extension of the basic model of virus dynamics introduced above. 

The experimental and theoretical results indicate that less virulent strains are more efficient in outcompeting the virulent ones in coinfected cells. Therefore, the fitness of variants of different virulence is density-dependent [168]. The cell competition model offers an explanation of several previous observations of suppression of high fitness mutants in dissimilar viral systems [169-172]. This density-dependent selection due to varying efficiency of viral replication alone or in coinfection is reminiscent of the concept of a competition-colonization trade-off in ecology [173]. Here, virulent viral variants play the role of colonizers and viruses efficient within coinfected cells are competitors. The attenuating effect of competitor-colonizer competition appears even more pronounced if many viruses from a broad spectrum of virulence are considered.

## Conclusions

Viral evolution and the genetic diversity it produces are fundamental factors for the success of vaccine candidates, because immune responses need to be stimulated against a potentially very broad spectrum of existing viruses and new viral immune escape variants are likely to be generated. Mathematical modeling of viral evolutionary dynamics will therefore play an increasingly important role in vaccine design. It can identify genomic regions that are under selective pressure, support the selection or construction of vaccine strains, predict evolutionary escape from immune pressure, guide vaccination campaigns, estimate the effect of therapeutic vaccines, and support the design of new attenuation strategies. Most of our discussion has been in the context of RNA viruses and many issues are most pronounced for this class of viruses. Nevertheless, we expect most statistical models and computational methods to be applicable to other viruses and different pathogens, too. On the other hand, the distinct evolutionary dynamics of influenza A and HIV-1, two of the most widely studied RNA viruses, have highlighted the need for careful analysis of viral infection dynamics within and among individuals.         

## List of abbreviations used

**HIV**: human immunodeficiency virus; **HCV**: hepatitis C virus; **HVB**: hepatitis B virus; **RSV**: respiratory syncytial virus; **SIV**: simian immunodeficiency virus; **FMDV**: foot-and-mouth disease virus; **Ad5**: adenovirus serotype 5 vector; **LAV**: live attenuated vaccine; **IV**: inactivated vaccine; **NA**: neuraminidase; **HA**: hemagglutinin; **MHC**: major histocompatibility complex; **UPGMA**: Unweighted Pair Group Method with Arithmetic mean; **MRCA**: most recent common ancestor; **CTL**: cytotoxic T lymphocyte; **HLA**: human leukocyte antigen; **LD**: linkage disequilibrium; **PDNs**: phylogenetic dependency networks; **CBNs**: conjunctive Bayesian networks; **ODE**: ordinary differential equations; **HI**: herd immunity.

## Competing interests

The authors declare no competing interests.

## Authors’ contributions

SO and NB wrote the paper.
